# Pituitary Apoplexy Complicated by Bilateral Anterior Cerebral Artery Territory Infarctions and Optic Tract Edema: A Case Report

**DOI:** 10.7759/cureus.107002

**Published:** 2026-04-13

**Authors:** Biju Pappachan, Saabh I Khalil, Abdulrahman J Aakef, Ryan Darayseh, Abdullah O Al Ani

**Affiliations:** 1 Radiology, Sheikh Khalifa Medical City, Ajman, ARE; 2 Medicine, Ajman University, Ajman, ARE

**Keywords:** acute cerebral infarction, magnetic resonance imaging, non-secreting pituitary macroadenoma, optic tract, pituitary apoplexy

## Abstract

Pituitary adenomas are common intracranial extra-axial neoplasms, classified based on size into microadenomas (<1 cm) and macroadenomas (>1 cm), and according to hormonal activity into functional and non-functional adenomas. Functional adenomas present with hormone-related syndromes, whereas non-functional macroadenomas typically cause symptoms due to mass effect, most commonly visual field defects. Uncommonly, pituitary adenomas may present acutely as pituitary apoplexy (PA), a medical emergency requiring prompt diagnosis and management to prevent mortality and long-term morbidity. Cerebral infarction secondary to compression of the anterior cerebral arteries is a rare complication of PA, and edema of the optic tracts is an even rarer imaging finding. We report a unique case of pituitary macroadenoma with apoplexy complicated by the simultaneous occurrence of bilateral anterior cerebral artery territory infarctions and symmetrical edema of the bilateral optic tracts. To the best of our knowledge, based on a structured literature search of PubMed, Embase, and Google Scholar, the coexistence of these two rare complications in PA has not been previously reported. This case highlights the critical role of cross-sectional imaging in identifying uncommon but serious vascular and optic pathway complications of PA, which have important implications for prognosis and management.

## Introduction

Pituitary adenomas are common intracranial extra-axial neoplasms, accounting for approximately 10-15% of primary central nervous system tumors, and are classified based on size into microadenomas and macroadenomas, as well as by hormonal activity into functioning and non-functioning tumors [1]. Non-functioning pituitary macroadenomas most commonly present with symptoms related to mass effect on adjacent structures, particularly visual field defects due to optic chiasmal compression [1,2].

Pituitary apoplexy (PA) is an uncommon but potentially life-threatening complication of pituitary adenomas, characterized by acute hemorrhage or infarction within the tumor. Rarely, PA may be complicated by cerebral ischemia due to vascular compression, vasospasm, systemic hypotension, or direct tumor-related vascular compromise of adjacent cerebral vessels [2]. We report a unique case of PA associated with simultaneous bilateral anterior cerebral artery territory infarctions and optic tract edema, highlighting an unusual and previously unreported combination of complications. PA complicated by cerebral infarction represents a rare but severe clinical entity associated with increased morbidity and diagnostic complexity. The simultaneous involvement of vascular territories and optic pathways remains poorly characterized, highlighting the importance of this case in expanding current understanding.

## Case presentation

A 37-year-old woman presented to the emergency department with a reduced level of consciousness, requiring urgent neurological evaluation. On initial evaluation, she was found to have sepsis, severe dehydration, and acute kidney injury, with raised inflammatory markers. There was no significant past medical or surgical history to account for her presentation. Laboratory investigations revealed hyponatremia and reduced levels of adrenocorticotropic hormone, prolactin, and other pituitary hormones, consistent with hypopituitarism.

**Figure 1:**
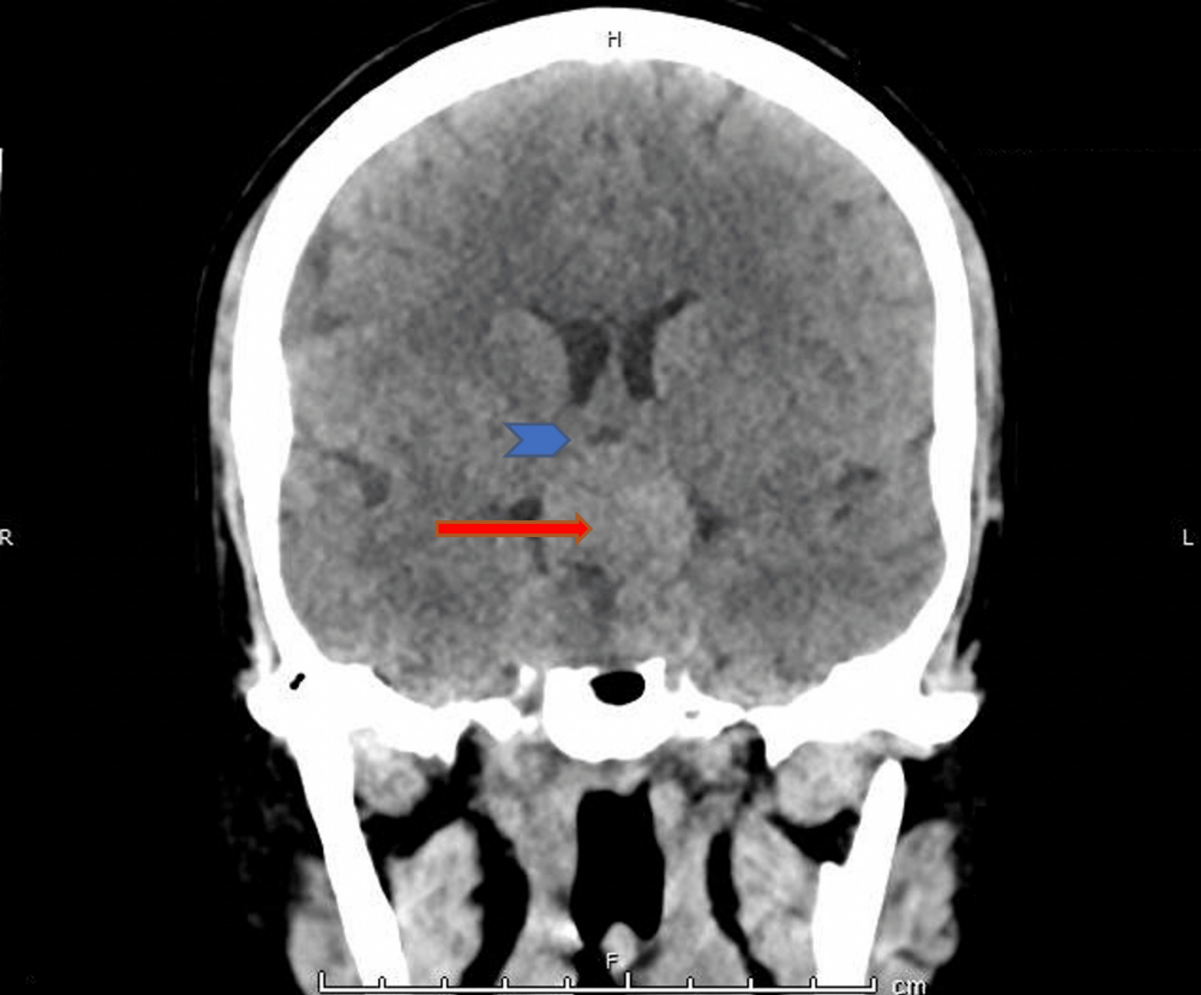
Non-contrast computed tomography (CT) coronal section at presentation showing an isodense sellar-suprasellar mass (arrow) causing compression of the optic chiasm and optic tracts (arrowheads).

A non-contrast computed tomography (CT) scan of the head demonstrated a well-defined sellar-suprasellar isodense, non-calcified soft tissue mass measuring approximately 35 × 27 × 25 mm, causing expansion of the sella turcica with erosion of the sellar floor and compression of the optic chiasm and optic tracts, findings suggestive of a pituitary macroadenoma (Figure 1). Intravenous contrast was not administered due to renal dysfunction. The patient was managed conservatively with intravenous fluids, empirical broad-spectrum antibiotics, and stress-dose corticosteroids for suspected PA. Close neurological and visual monitoring was undertaken. Surgical decompression was not pursued, as there was no evidence of progressive visual impairment or neurological deterioration, and the patient demonstrated clinical stabilization with medical therapy.

**Figure 2:**
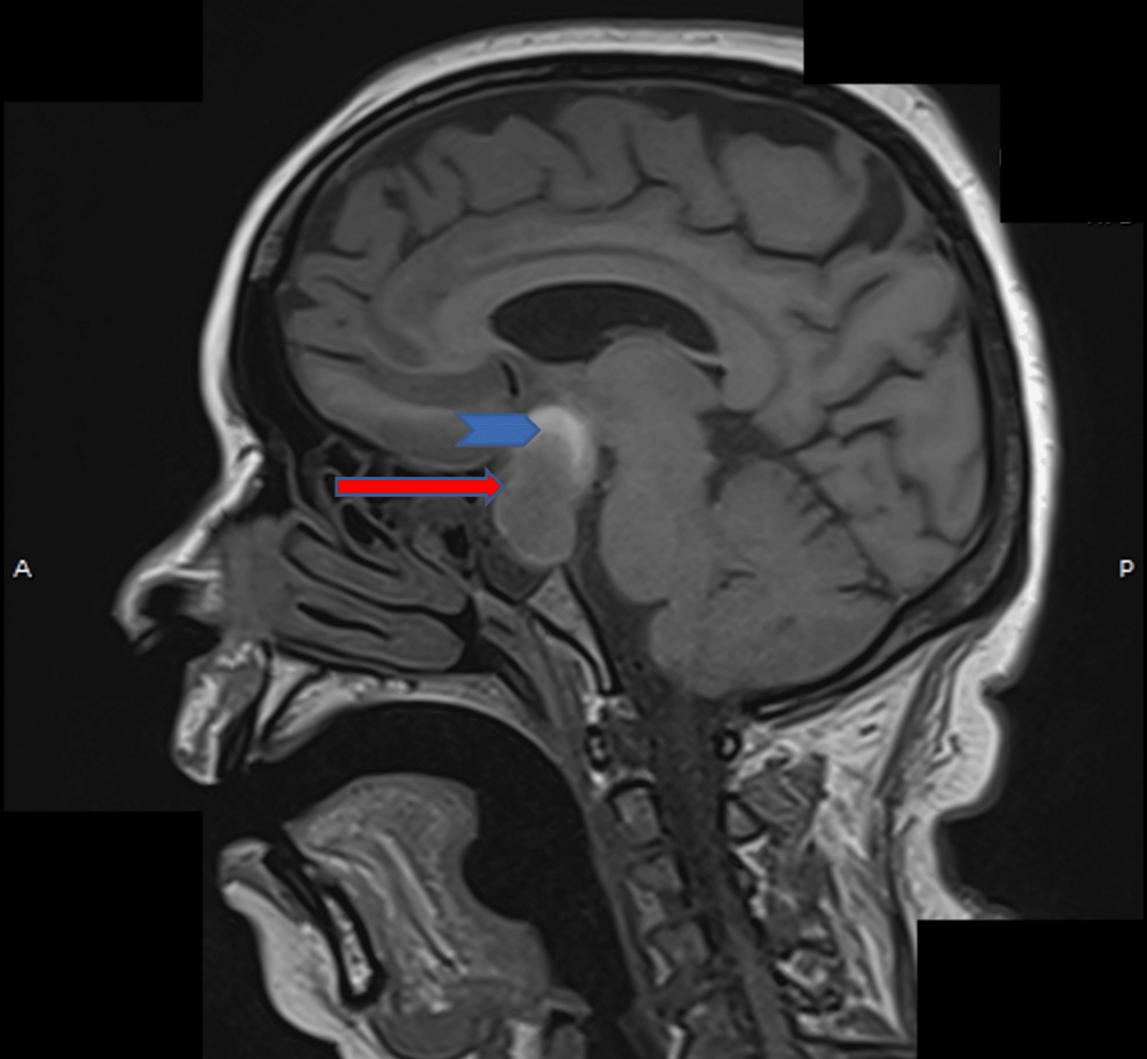
Sagittal T1-weighted MRI demonstrating a heterointense sellar-suprasellar mass with a characteristic “figure-of-eight” configuration (arrow) and hemorrhagic component in the superior portion of the lesion (arrowhead).

**Figure 3:**
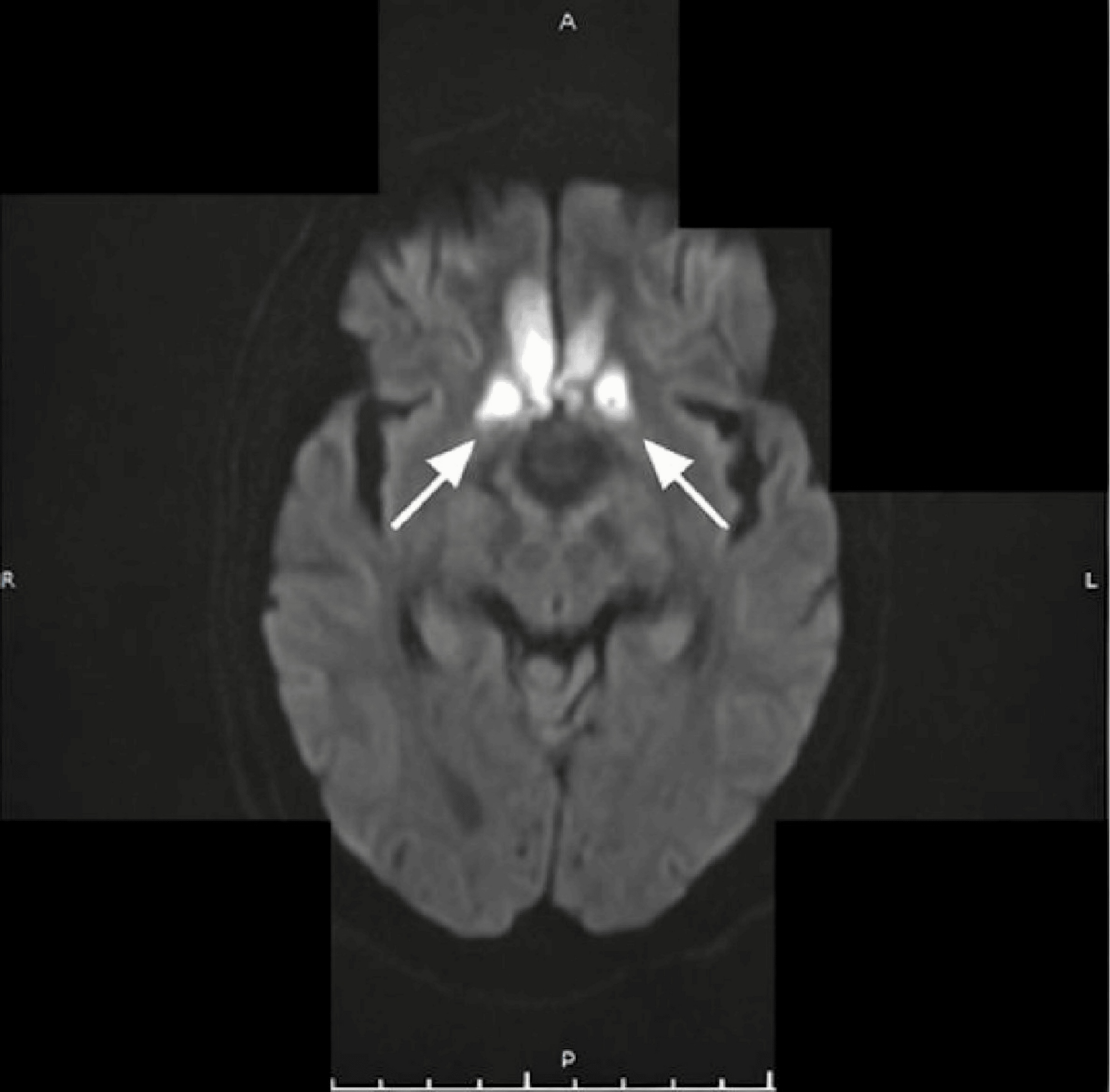
Axial diffusion-weighted imaging (DWI) obtained eight days after presentation demonstrating symmetrical areas of diffusion restriction in the bilateral basifrontal regions (arrows), consistent with bilateral anterior cerebral artery territory infarctions.

**Figure 4:**
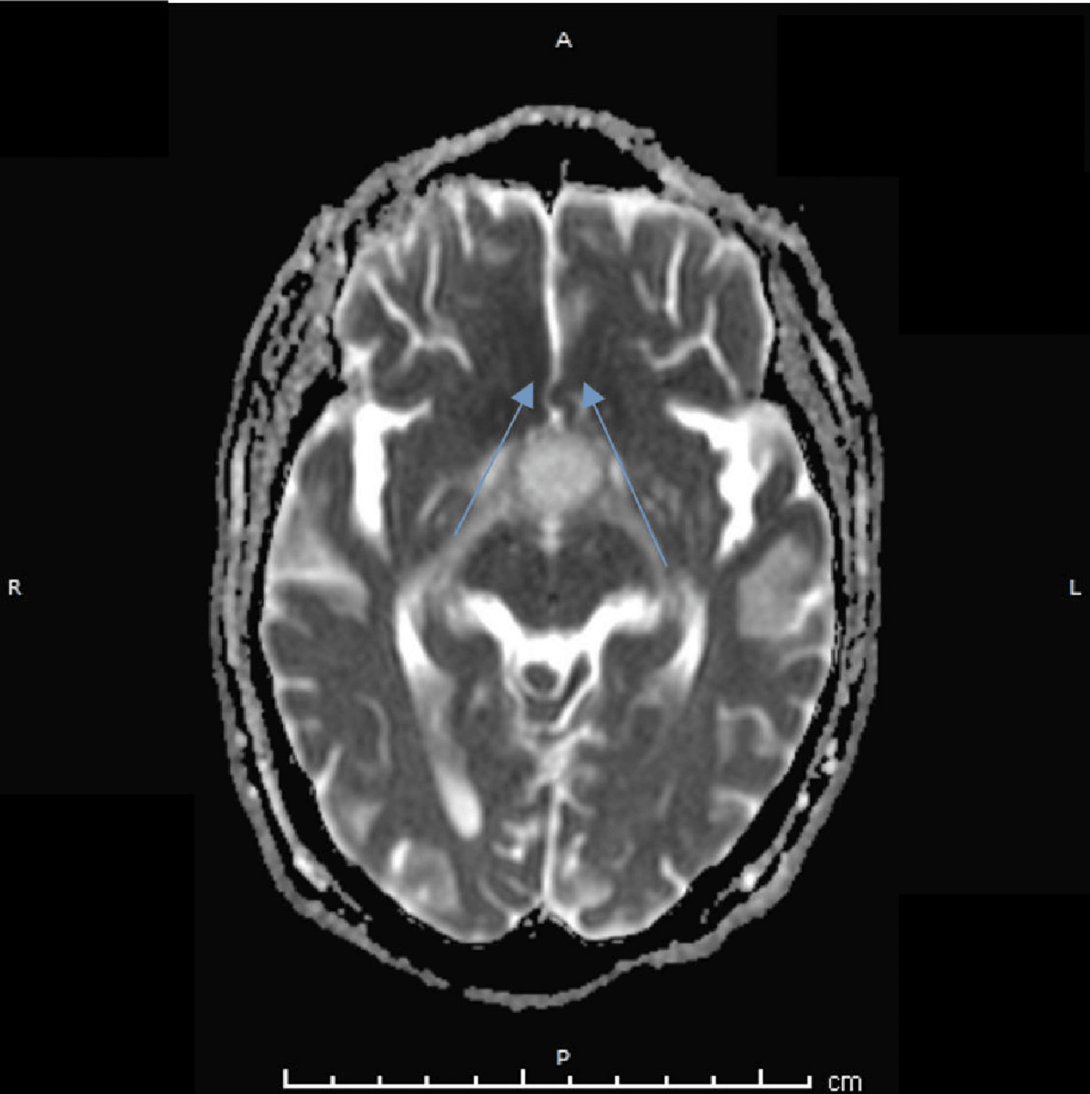
Axial apparent diffusion coefficient (ADC) map demonstrating a bilateral hypointense signal within the basifrontal regions (arrows), consistent with true diffusion restriction in keeping with acute bilateral anterior cerebral artery (ACA) territory infarctions.

**Figure 5:**
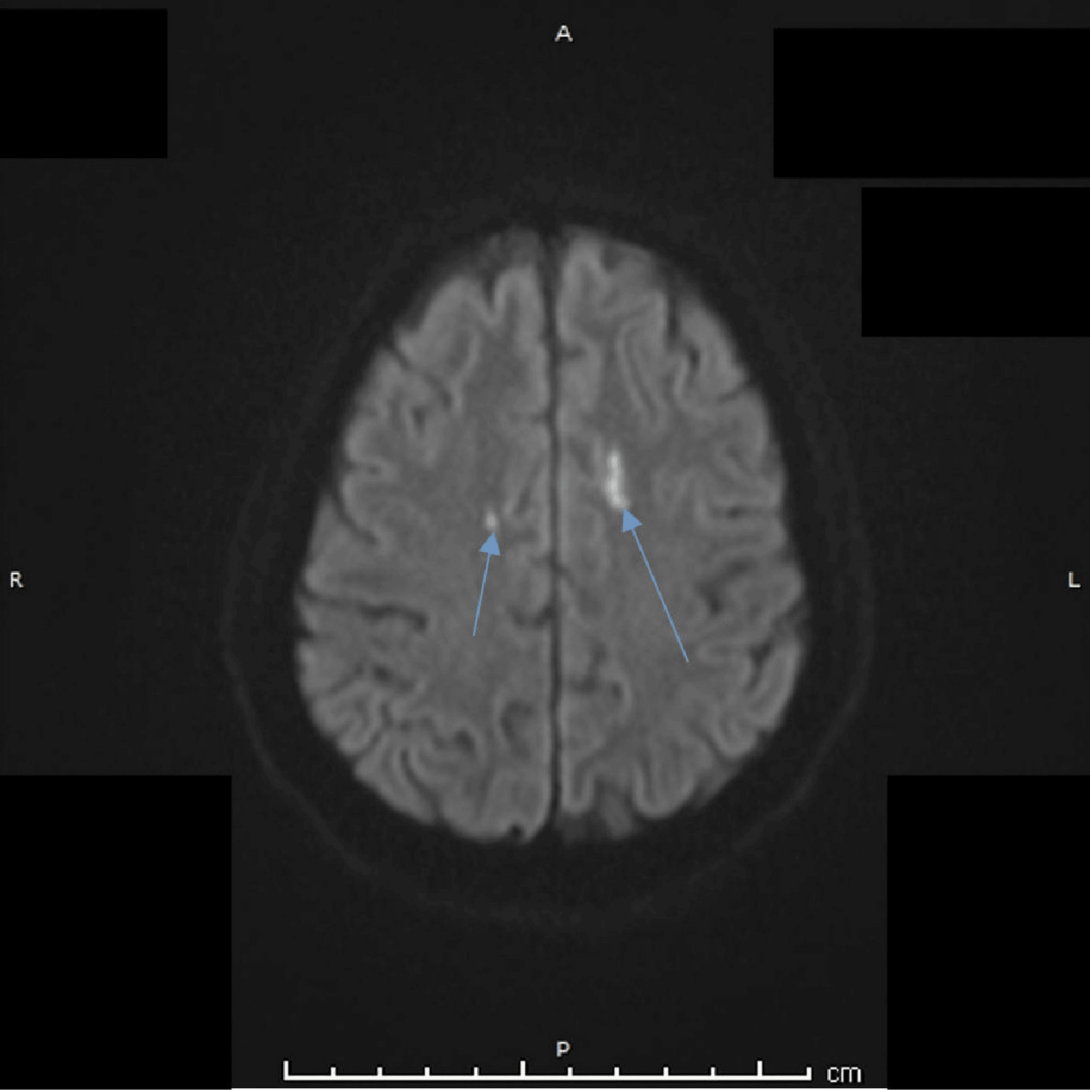
Axial diffusion-weighted imaging (DWI) obtained eight days after presentation demonstrating a bilateral hyperintense signal within the high frontal regions (arrows), consistent with diffusion restriction in keeping with bilateral anterior cerebral artery (ACA) territory infarctions.

A non-contrast magnetic resonance imaging (MRI) study performed one week later revealed a heterointense sellar-suprasellar mass with a characteristic “figure-of-eight” configuration. Hemorrhage was noted within the superior portion of the lesion, appearing hyperintense on T1-weighted images and showing blooming on susceptibility-weighted imaging, consistent with PA (Figure 2). The lesion could not be clearly separated from the pituitary gland. Diffusion-weighted imaging (DWI) and apparent diffusion coefficient (ADC) maps showed no diffusion restriction within the pituitary lesion; however, diffusion restriction was identified in the bilateral basifrontal and high frontal lobes on DWI with corresponding low signal on ADC maps, consistent with bilateral anterior cerebral artery territory infarctions (Figures 3-5). Neurological examination was limited due to the patient’s reduced level of consciousness; however, no clearly lateralizing focal neurological deficits were documented at the time of assessment. Formal neuro-ophthalmological assessment, including visual acuity, visual field testing, and fundoscopic examination, was not feasible at the time of presentation due to the patient’s reduced level of consciousness and critical clinical status.

**Figure 6:**
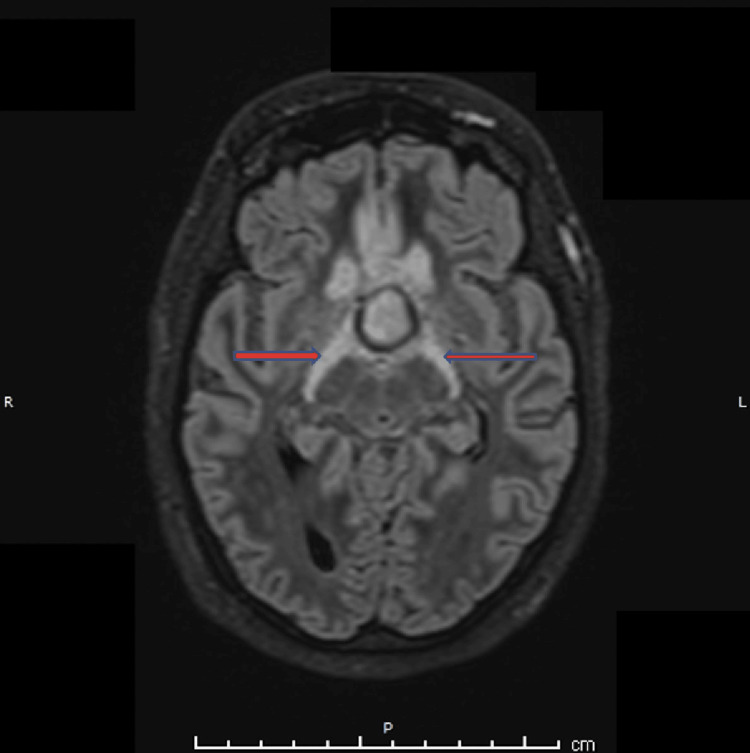
Axial 3D SPACE FLAIR image demonstrating a sellar–suprasellar pituitary macroadenoma with symmetrical hyperintensity along the bilateral optic tracts (arrows), consistent with optic tract edema (“moustache sign”). FLAIR: Fluid-attenuated inversion recovery; SPACE: Sampling Perfection with Application optimized Contrasts using different flip angle Evolution

**Figure 7:**
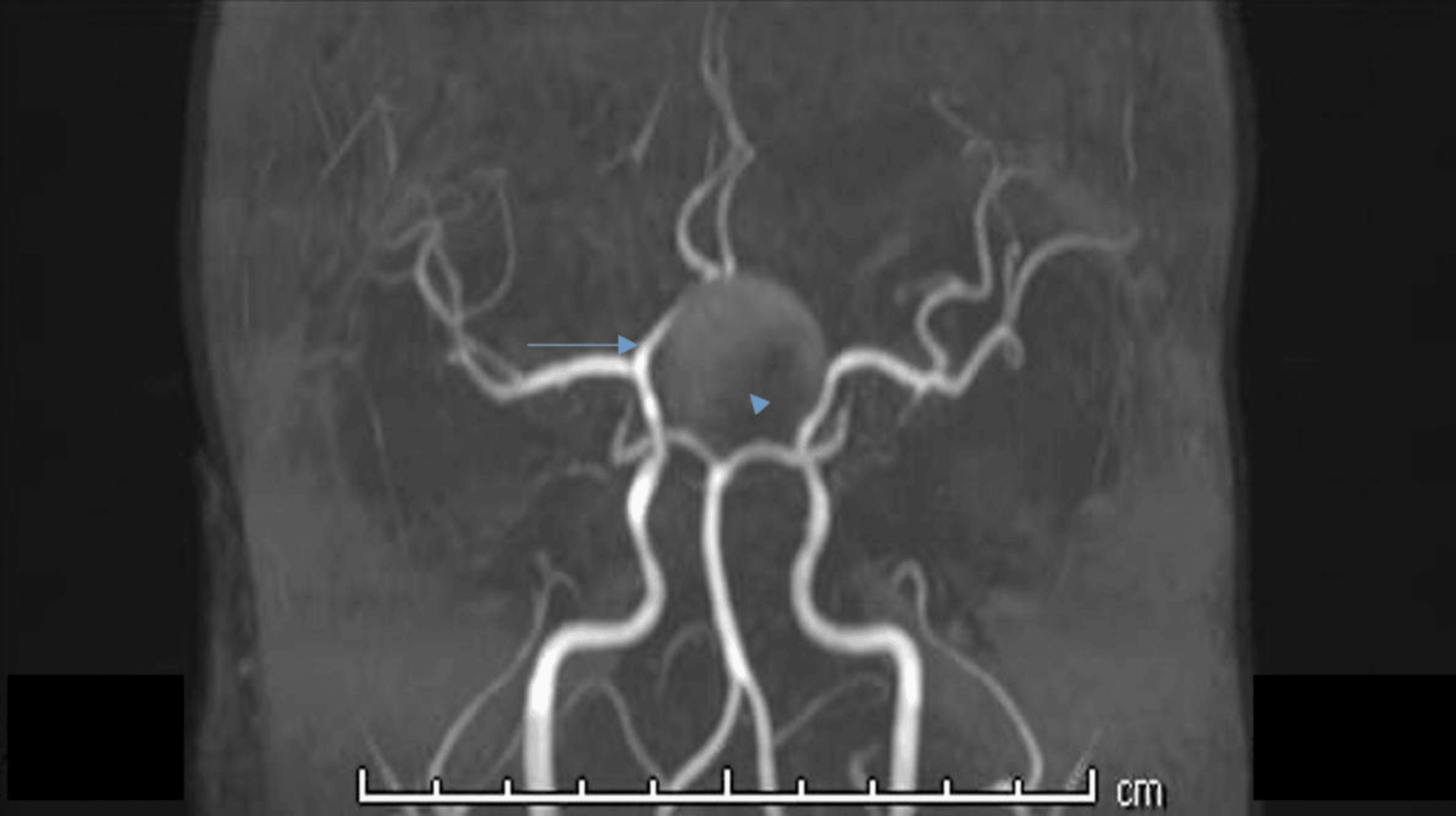
Coronal 3D time-of-flight magnetic resonance angiography (TOF-MRA) reconstruction demonstrating a sellar–suprasellar mass (arrowhead) causing splaying of the circle of Willis, with compression and narrowing of the proximal A1 segments of the bilateral anterior cerebral arteries (arrows).

Symmetrical edematous signal changes were observed along the bilateral optic tracts, producing a characteristic “moustache” appearance, with facilitated diffusion on DWI (Figure 6). On T2-weighted and susceptibility-weighted sequences, the pituitary lesion was marginated by a smooth hypointense rim. No evidence of subarachnoid hemorrhage was identified. Three-dimensional time-of-flight magnetic resonance angiography (TOF-MRA) demonstrated patent internal carotid arteries (ICAs) with splaying of the circle of Willis and compression with resultant narrowing of the bilateral A1 segments of the anterior cerebral arteries, more pronounced on the left side (Figure 7).

**Figure 8:**
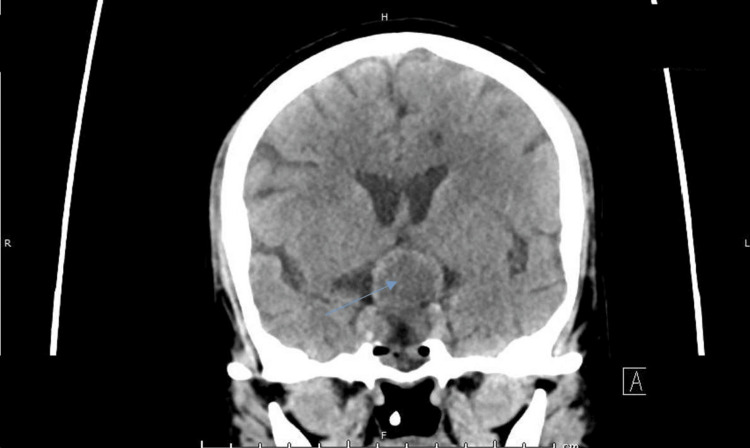
Follow-up non-contrast computed tomography (NCCT) of the brain in the coronal plane obtained 10 days after presentation demonstrating interval cystic degeneration of the previously identified sellar–suprasellar pituitary macroadenoma (arrow).

Due to clinical deterioration, a follow-up non-contrast CT scan demonstrated interval cystic (hypodense) transformation of the pituitary lesion, along with bilateral parasagittal basifrontal infarctions (Figure 8). The patient was subsequently referred to a higher-level center for endoscopic transsphenoidal hypophysectomy. Postoperative and long-term neurological and endocrine outcomes were not available, representing a limitation of this case.

## Discussion

Pituitary adenomas are common intracranial neoplasms, accounting for approximately 12.7% of primary central nervous system tumors, and are particularly prone to hemorrhage, infarction, or necrosis due to their unique vascular supply [2-5]. PA represents an acute clinical syndrome resulting from hemorrhage or infarction within the gland, occurring in approximately 2.3-7.5% of adenomas and may be the first presentation of an otherwise undiagnosed tumor [6-8].

Although PA is well recognized, its association with cerebral infarction is rare, and when present, it reflects a more severe clinical entity [2,3]. Ischemic complications may arise through several mechanisms, including mechanical compression of adjacent vascular structures, vasospasm secondary to vasoactive substances, tumor invasion, or systemic hypotension [2,3,9]. Among these, mechanical compression of the anterior circulation, particularly the anterior cerebral artery (ACA) or ICA, is the most frequently implicated mechanism [9,10].

In the present case, imaging findings strongly support mechanical compression of the ACA segments, demonstrated by splaying of the circle of Willis on TOF-MRA and the development of bilateral ACA territory infarctions. This pattern is characteristic, as the ACA courses in close proximity to the suprasellar region and is particularly vulnerable to mass effect, whereas the middle cerebral artery is relatively spared due to its lateral anatomical course [10].

Neuroimaging plays a central role in diagnosis and pathophysiological characterization. CT is typically the first-line modality in acute settings, allowing rapid identification of a sellar-suprasellar mass and exclusion of other acute intracranial pathologies such as subarachnoid hemorrhage [5]. However, MRI provides superior sensitivity and specificity for both pituitary apoplexy and associated cerebral infarction [3,8]. DWI demonstrates early ischemic changes as restricted diffusion with a corresponding low signal on ADC maps [5,11,12]. In this case, DWI and ADC sequences confirmed bilateral ACA infarctions, while follow-up imaging demonstrated cystic degeneration of the macroadenoma, reflecting the dynamic evolution of apoplexy [5,8].

An additional notable feature in this case is optic tract edema, an uncommon but increasingly recognized imaging finding in pituitary macroadenomas. This finding, often referred to as the “moustache sign,” is thought to result from impaired cerebrospinal fluid dynamics and interstitial fluid accumulation due to obstruction of perivascular (Virchow-Robin) spaces at the suprasellar region [11-13]. While impaired cerebrospinal fluid dynamics and interstitial fluid accumulation related to obstruction of perivascular (Virchow-Robin) spaces are proposed mechanisms, alternative explanations include direct compressive axonal injury and retrograde transsynaptic degeneration secondary to chronic optic chiasm compression. Its presence reflects a significant mass effect and has important implications for visual pathway involvement.

Endocrine dysfunction is present in the majority of patients with PA, affecting up to 80% of cases, with adrenocorticotropic hormone deficiency being the most clinically significant [4]. Associated electrolyte disturbances, including hyponatremia, may occur due to secondary adrenal insufficiency and impaired vasopressin regulation [14].

From a management perspective, PA complicated by cerebral infarction carries a poorer prognosis compared to isolated apoplexy [3]. In the present case, initial conservative management was selected due to the absence of progressive visual or focal neurological deficits at presentation. The subsequent clinical deterioration underscores the dynamic nature of pituitary apoplexy and raises the possibility that earlier surgical intervention may have altered the clinical course. However, at the time of initial assessment, there were no definitive indications for urgent decompression, and the management approach was consistent with existing clinical practice. Early recognition and prompt referral for neurosurgical evaluation are critical, as urgent decompression in selected patients has been associated with improved neurological and endocrine outcomes [3,7]. Long-term follow-up with serial MRI is recommended to monitor for tumor recurrence, typically at 3-6 months initially, followed by annual imaging [15].

## Conclusions

PA is an unpredictable and potentially life-threatening condition that requires prompt recognition and multidisciplinary management. Cross-sectional imaging is essential for identifying associated vascular and optic pathway complications. In this case, initial conservative management was followed by clinical deterioration requiring referral for surgical intervention, underscoring the importance of close monitoring and timely escalation of care. Although postoperative and long-term follow-up data were unavailable, this case highlights the dynamic clinical course of PA and the need for individualized management strategies.
